# Fatal Liver and Bone Marrow Toxicity by Combination Treatment of Dichloroacetate and Artesunate in a Glioblastoma Multiforme Patient: Case Report and Review of the Literature

**DOI:** 10.3389/fonc.2016.00204

**Published:** 2016-10-07

**Authors:** Martin Uhl, Stefan Schwab, Thomas Efferth

**Affiliations:** ^1^Department of Neurology, University of Erlangen-Nuremberg, Erlangen, Germany; ^2^Institute of Pharmacy and Biochemistry, Johannes Gutenberg University, Mainz, Germany

**Keywords:** adverse side effects, cancer, chemotherapy, toxicology

## Abstract

A 52-year-old male patient was treated with standard radiochemotherapy with temozolomide for glioblastoma multiforme (GBM). After worsening of his clinical condition, further tumor-specific treatment was unlikely to be successful, and the patient seeked help from an alternative practitioner, who administered a combination of dichloroacetate (DCA) and artesunate (ART). A few days later, the patient showed clinical and laboratory signs of liver damage and bone marrow toxicity (leukopenia, thrombocytopenia). Despite successful restoration of laboratory parameters upon symptomatic treatment, the patient died 10 days after the infusion. DCA bears a well-documented hepatotoxic risk, while ART can be considered as safe concerning hepatotoxicity. Bone marrow toxicity can appear upon ART application as reduced reticulocyte counts and disturbed erythropoiesis. It can be assumed that the simultaneous use of both drugs caused liver injury and bone marrow toxicity. The compassionate use of DCA/ART combination therapy outside of clinical trials cannot be recommended for GBM treatment.

## Introduction

Glioblastoma multiforme (GBM) is an aggressive brain tumor that is currently treated with a combination of radiotherapy and temozolomide (TMZ) chemotherapy. The prognosis is unfavorable with an average survival of 15 months (1–3). In this desperate situation, it is not uncommon for patients to seek help outside standard medicine from alternative practitioners and healers. Often, non-approved remedies or unproven combination of drugs are prescribed, which occasionally may lead to undesired side effects or even life-threatening toxicities.

Dichloroacetate (DCA) is generated as by-product of chlorination of drinking water and by metabolitzation of drugs and chemicals (4). DCA accumulation in groundwater is considered as potential health hazard. *In vitro* and *in vivo* investigations showed that DCA inhibits tumor growth by redirecting glycolysis to oxidative phosphorylation and oxidative removal of lactate *via* pyruvate (5). Although five GBM patients have been previously treated with DCA (6), there is only limited knowledge about the efficacy or toxicity of DCA in cancer therapy.

In addition to their antimalarial activity, the artemisinin (ARS) derivatives [artesunate (ART), artemether, dehydroartemisinin] also exert anticancer activity *in vitro* and *in vivo* (7–13), including some brain tumor models (14–18). Compassionate use of ARS-type drugs encouraged the initiation of phase I/II trials in cancer patients (19–27). Most of these studies report are case reports or consist of only small numbers of patients. Therefore, there is still limited evidence regarding the safe use of ARS in cancer patients.

In the present case report, we describe a patient, who died with severe liver and bone marrow toxicity after intake of combined DCA and ART.

## Case Report

A 52-year-old male patient was diagnosed with GBM after suffering for several weeks from cognitive decline, headaches, gait ataxia, and a series of epileptic seizures. The initiation of adjuvant therapy was delayed by complicated wound healing, but finally – 53 days after surgery – radiotherapy up to 60 Gy of the tumor region was initiated with simultaneous TMZ chemotherapy (75 mg/m^2^) according to local guidelines (28).

The general state of health was unfavorable (Karnofsky score: 50). The patient suffered from right-side hemiparesis and required considerable help and medical assistance. Therefore, adjuvant TMZ chemotherapy was ruled out, and rehabilitation actions were initiated. Rehabilitation had to be discontinued 128 days after surgery, because of another series of epileptic seizures. Antiepileptic treatment was escalated to 1800 mg valproic acid (VA), 3000 mg levetiracetam, 200 mg lacosamide, and 20 mg clobazam. Progressive intracranial tumor burden by CT and Fet-PET scan diagnosis was considered as non-suitable for tumor-specific treatment, and steroid medication was escalated.

**Figure 1:**
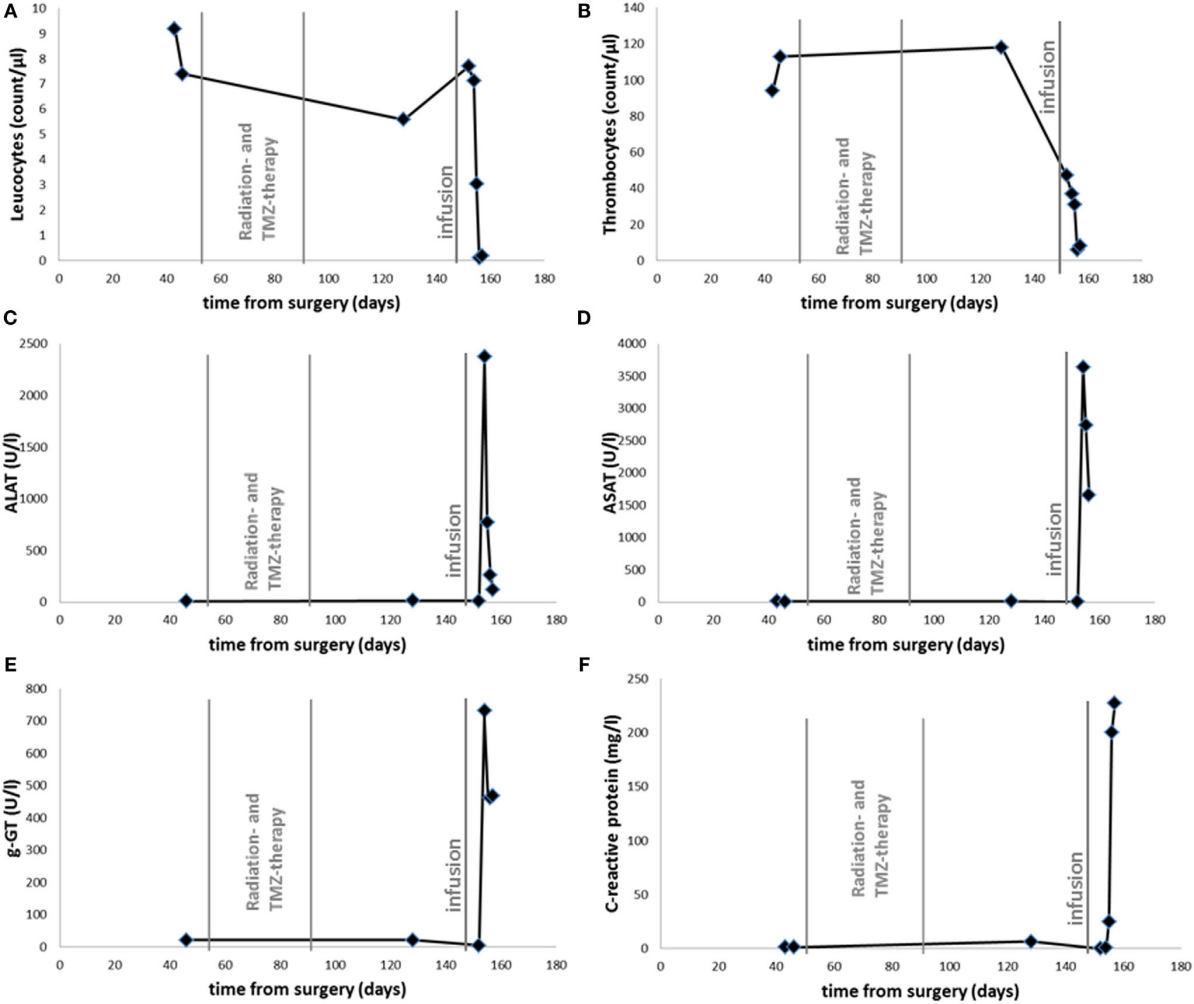
**Time course of leukocyte (A), thrombocyte (B) count, serum levels of ALAT (C), ASAT (D), g-GT (E), and CRP (F)**. Radiotherapy with Temozolomide as indicated between 53 and 92 days after surgery. Infusion with ART and DCA is labeled 148 days after surgery.

At that point, the patient and his family were seeking help from an alternative practitioner. An unknown amount of DCA was administered and ART (2.5 mg/kg bodyweight) was intravenously infused 148 days after surgery. At that time, the patient had a stable/unchanged concomitant medication. The patient’s cognitive condition declined during the following days with adynamia, severe headaches, and psychomotoric retardation in rapid change with signs of delusions. After admission to the hospital, epileptic activity was not found by EEG and CT scanning did not show relevant changes concerning mass effect or edema. However, blood examinations showed signs of exsiccosis, pancytopenia, and markedly increased hepatic enzyme activities (Figure 1). Upon fluid substitution, laboratory parameter stabilized. However, two days after hospitalization, the state of the patient suddenly deteriorated with hypotension, systemic signs of infection, and a series of epileptic seizures. Discussing the need for intensified medical intervention and possible mechanical ventilation, the family did not wish these the actions to be undertaken according to the patient’s provision. The patient died during the course of the following night and 157 days after surgery.

The timing of events can be summarized as follows:

- Surgery at day 0
- Start of radiotherapy 53 days after surgery
- End of radiotherapy 92 days after surgery
- Infusion of ART and DCA 148 days after surgery
- First signs of toxicity 154 days after surgery (elevated liver enzymes and hematotoxicity)
- Death of the patient 157 days after surgery

A valuable measure for the causality of adverse reactions of drugs in patients with liver injury is the Roussel Uclaf Causality Assessment Method (RUCAM) (29, 30). RUCAM considers all relevant criteria for liver injury by drugs. We applied the RUCAM scoring system to the patient presented here and found an overall quantitative grading of causality of 6, which indicates reasonable probability that the combinational administration of DCA and ART caused liver injury (Table 1).

**Table 1:** **Causality assessment of adverse reactions to the DCA/ART combination treatment according to RUCAM (29, 30)**.

Criterion	Observation	Given score	Score range
1. Time to onset of the reaction	Toxic reaction 6 days after treatment	2	(+1 to +2)
2. Course of the reaction	Decrease <50% within 30 days	3	(−2 to +3)
3. Risk factors for drug reaction	Age of patient ≥55 years	0	(0 to +1)
4. Concomitant drugs	No information	0	(−3 to 0)
5. Non-drug-related causes	HAV, HBV, and HCV serology missing, no biliary obstruction, no alcoholism, no hypotension	0	(−3 to +2)
6. Previous information on the drug	Hepatotoxicity published, but unlabeled	1	(0 to +2)
7. Response to readministration	Not possible, because patient died	0	(−2 to +3)

Total		6	

*Quantitative grading of causality: ≤0, excluded; 1–2, unlikely; 3–5, possible; 6–8 probable; ≥9, highly probable*.

## Discussion

The severity and outcome of this case of compassionate use of alternative medication is remarkable. While the hepatotoxic potential of DCA is well documented, ART is actually considered a rather safe antimalarial drug. It can be speculated that the specific combination of both drugs provoked fatal liver and bone marrow toxicity in the patient.

At the day of hospitalization, prior alternative medication had not been declared by the patient. Therefore, liver toxicity by VA or TMZ has been suspected. In the past, severe and even fatal toxicity were reported for both for VA (31–36) and for TMZ (37–40). Taking into account the additional sudden decline in leukocyte and thrombocyte counts during the next days and considering the prior normal values made this possibility, however, rather unlikely. The dynamics of TMZ- or VA-caused liver damage usually represent more continuous processes. The nadir of TMZ is expected after 21 days. Even delayed forms of bone marrow toxicity are not comparable to the dramatic decline observed here.

The cause of death remains speculative, since an autopsy was not performed in accordance to the patient’s provision and family wishes. We consider aspiration pneumonia or spontaneous internal bleeding as possible causes for the sudden decline of blood pressure.

As shown in Table 2, DCA administration in animal experiments induced hepatotoxicity and hepatocarcinogenesis. DCA increased hepatic oxidative stress and disturbed liver metabolism. Although treatment of five GBM patients with DCA did not reveal hepatotoxicity (6), there is evidence from preclinical *in vivo* experiments that DCA affects the liver (Table 2) (4, 41). However, a straightforward conclusion to the observed hepatotoxicity in the present case is difficult, because the dose of applied DCA to the patient was not disclosed by the alternative practitioner.

**Table 2:** **Literature survey on hepatotoxicity by DCA *in vivo***.

Experimental model	Treatment dose	Route of administration	Duration of treatment	Effect	Reference
Dogs	300 mg/kg	Intravenously	1 h	Decrease of tissue lactate levels in liver	(42)
B6C3F1 mice	1–2 g/L	Drinking water	52 weeks	Enlarged livers, cytomegaly, and glycogen accumulation	(43)
B6C3F1 and Swiss-Webster mice	300–2000 mg/L	Drinking water	14 days	Tumorigenesis is influenced by necrosis and reparative hyperplasia, increased ^3^H-thymidine labeling index	(44)
B6C3F1 mice	200–600 mg/L	Drinking water	72 h	Markedly enlarged liver, cytomegaly, glycogen accumulation, recurrent liver necrosis with high proliferation rates, peroxisome induction, and lipofuscin accumulation	(45)
B6C3F1 mice	2.0 g/L	Drinking water	38 or 50 weeks	Induction of hepatocellular lesions with increased cell divisions; increased c-Jun/c-Fos expression	(46)
B6C3F1 mice	0.5 g/L	Drinking water	2 weeks	4-fold increase of *in vitro* colony formation of hepatocytes suggesting promotion of clonal expansion of anchorage-independent hepatocytes *in vivo*	(47)
B6C3F1 mice	2 g/L	Drinking water	48 weeks	Increase of tumor growth rates	(48)
B6C3F1 mice	0.2–3 g/L	Drinking water	4–12 weeks	Increase of glycogen concentration in liver	(49)
B6C3F1 mice	0.1–2 g/L	Drinking water	2–10 weeks	Reduction of serum insulin, downregulation of insulin receptor, and increased MAP kinase phosphorylation	(50)
B6C3F1 mice	0.5 or 2 g/L	Drinking water	35–52 weeks	Induction of liver tumors, which were c-Jun-positive	(51)
Fischer-344 rats	0.05–20 mg/kg	Intravenously or by gavage	7 days	Oral bioavailability was 0–13% in control rats and 14–75% in GSTZ-depleted rats	(52)
Sprague-Dawley rats	2.5 μg–50 mg/kg/day	Drinking water	12 weeks	GSTZ1-1 activity and expression decreased to 95–100% and recovered 8 weeks after cessation	(53)
B6C3F1 mice	300 mg/kg	By gavage	6 or 12 h	Increased production of superoxide anion, lipid peroxidation, and DNA-single strand breaks	(54)
B6C3F1 male mice	7.7–410 mg/kg/day	By gavage	4 or 13 weeks	Hepatomegaly at 410 mg/kg/day. Dose-dependent increase of SOD activity, lipid peroxidation, and DNA-single strand breaks	(55)
Sprague-Dawley rats	500 mg/kg/day	By gavage	8 weeks	Dechlorination of DCA was higher in cytosol than in mitochondria by GSTZ1	(56)
PKD rats	75 mg/L	Drinking water	8 weeks	Only male rats with polycystic kidney disease (PKD) showed increased disease severity (cystic enlargement and proteinuria)	(57)
B6C3F1 mice	7.5–30 mg/kg/day	By gavage	13 weeks	Dose-dependent increase of SOD production, lipid peroxidation and DNA-single strand breaks	(58)

The clinical safety of ART is well documented. Large clinical trials and meta-analyses of clinical trials dealing with many thousands of malaria patients did not unravel serious adverse effects (59, 60). Preclinical toxicity studies gave some hints for neurotoxicity, embryotoxicity, genotoxicity, hematotoxicity, cardiotoxicity, nephrotoxicity, and allergic reaction (61). Long-term application of low ARS concentrations may be more toxic than short-term application of high doses. This may explain, why toxicities can be observed in animal experiments, but not in human studies. A large meta-analysis with 5000 malaria patients revealed that hepatotoxicity was a rare event, and elevated liver enzymes have been found in 0.9% of all cases (59). Although most papers on clinical safety were published in the context of malaria treatment, there are also some reports on the use of ARS-derivatives in cancer patients. Case reports on the compassionate use of ART or artemether in patients, with laryngeal squamous cell carcinoma, uveal melanoma, pituitary macroadenoma, and prostate carcinoma, reported that the ARSs were well tolerated with no additional side effects in addition to those caused by standard chemotherapy. A randomized controlled trial with 120 advanced non-small cell lung cancer patients on vinorelbine alone versus vinorelbine plus ART did not find significant differences in toxicity between the two treatment groups (23). In a pilot phase I/II trial in 10 patients suffering from cervical carcinoma, artenimol reduced clinical symptoms, vaginal discharge, and pain, and no adverse events of grade 3 and 4 were observed (24). Another phase I/II pilot study in veterinary cancers was conducted in 23 dogs with non-resectable tumors. No neurological or cardiac toxicity was observed, and seven dogs exhibited no adverse effects at all. Fever and hematological or gastrointestinal toxicity, mostly transient, occurred in 16 dogs. One dog died from treatment-unrelated pneumonia (25). As reported from a randomized, double-blind placebo-controlled pilot study in 23 colorectal cancer patients, oral ART therapy was well tolerated without signs of hepatotoxicity (26). Another recent phase I trial on 23 metastasized breast cancer patients reported that four patients had adverse events of the auditory system possibly related to the intake of ART. However, none of these side effects were severe adverse events. Four patients had adverse events concerning the vestibular system, one of which was severe, but fully reversible after discontinuation of ART treatment (27). In summary, hepatotoxicity has not been found in any of these patients.

Hematotoxicity is worth mentioning in this context, because the patient suffered from reduced leukocyte and thrombocyte counts. The toxicity of ARS-type drugs on leukopoiesis is controversially discussed, and both enhanced and inhibited leukocyte functions have been observed (61). Dihydroartemisinin ameliorated inflammatory disease (62). However, ARS-derivatives exhibited higher cytotoxicity *in vitro* toward hematopoietic progenitor cells of the granulocyte-monocyte lineage (CFU-GM) than toward cancer cells (63), indicating that myelosuppression might be an issue in cancer therapy. While thrombocytopenia was apparently not relevant, damage of erythrocytes occurred in animal experiments (61). A sensitive measure for erythropoiesis is the blood count of reticulocytes in peripheral blood. Reduced reticulocyte counts (as erythrocyte precursors) have not only been observed *in vitro* and in animals, but also in human patients upon treatment with ARS-type drugs (59, 61, 64, 65).

In conclusion, the presented case illustrates the possible consequences of compassionate use of non-approved drugs or unproven drug combinations. Drug therapy should always be in accordance to the guidelines of good clinical practice.

## Author Contributions

MU and SS: treated the patient. TE: wrote the paper.

## Conflict of Interest Statement

The authors declare that the research was conducted in the absence of any commercial or financial relationships that could be construed as a potential conflict of interest.

## Abbreviations

ARS, artemisinin; ART, artesunate; DCA, dichloroacetate; GBM, glioblastoma multiforme; TMZ, temozolomide; VA, valproic acid.
